# Author Correction: One-Tip enables comprehensive proteome coverage in minimal cells and single zygotes

**DOI:** 10.1038/s41467-024-47496-x

**Published:** 2024-04-10

**Authors:** Zilu Ye, Pierre Sabatier, Javier Martin-Gonzalez, Akihiro Eguchi, Maico Lechner, Ole Østergaard, Jingsheng Xie, Yuan Guo, Lesley Schultz, Rafaela Truffer, Dorte B. Bekker-Jensen, Nicolai Bache, Jesper V. Olsen

**Affiliations:** 1 State Key Laboratory of Common Mechanism Research for Major Diseases, Suzhou Institute of Systems Medicine, Chinese Academy of Medical Sciences & Peking Union Medical College, Suzhou, China; 2 Novo Nordisk Foundation Center for Protein Research, University of Copenhagen, Copenhagen, Denmark; 3 Department of Surgical Sciences, Uppsala University, Uppsala, Sweden; 4 Core Facility for Transgenic Mice, Department of Experimental Medicine, University of Copenhagen, Copenhagen, Denmark; 5 Tecan Group Ltd., Männedorf, Switzerland; 6 Evosep Biosystems, Odense, Denmark

**Keywords:** Proteomic analysis, Proteomics

Correction to: *Nature Communications* 10.1038/s41467-024-46777-9, published online 20 March 2024

The original version of this Article contained an error in the first sentence of the Competing Interests statement, which incorrectly read ‘Yuan Guo, Lesley Schultz and Rafaela Truffer are employees of Tecan, the developers of the Uno Single Cell Sorter.’ The correct version replaces this sentence with ‘Yuan Guo, Lesley Schultz and Rafaela Truffer are employees of Tecan, the vendor of the Uno Single Cell Dispenser.’ This has been corrected in both the PDF and HTML versions of the Article.

