# Supplementary material for: One-Tip enables comprehensive proteome coverage in minimal cells and single zygotes
Source: Nat Commun. 2024 Mar 20;15:2474. doi: 10.1038/s41467-024-46777-9 (PMC10951212; doi:10.1038/s41467-024-46777-9)
Supplement: Supplementary file 1 — Supplementary Information [file 41467_2024_46777_MOESM1_ESM.pdf]

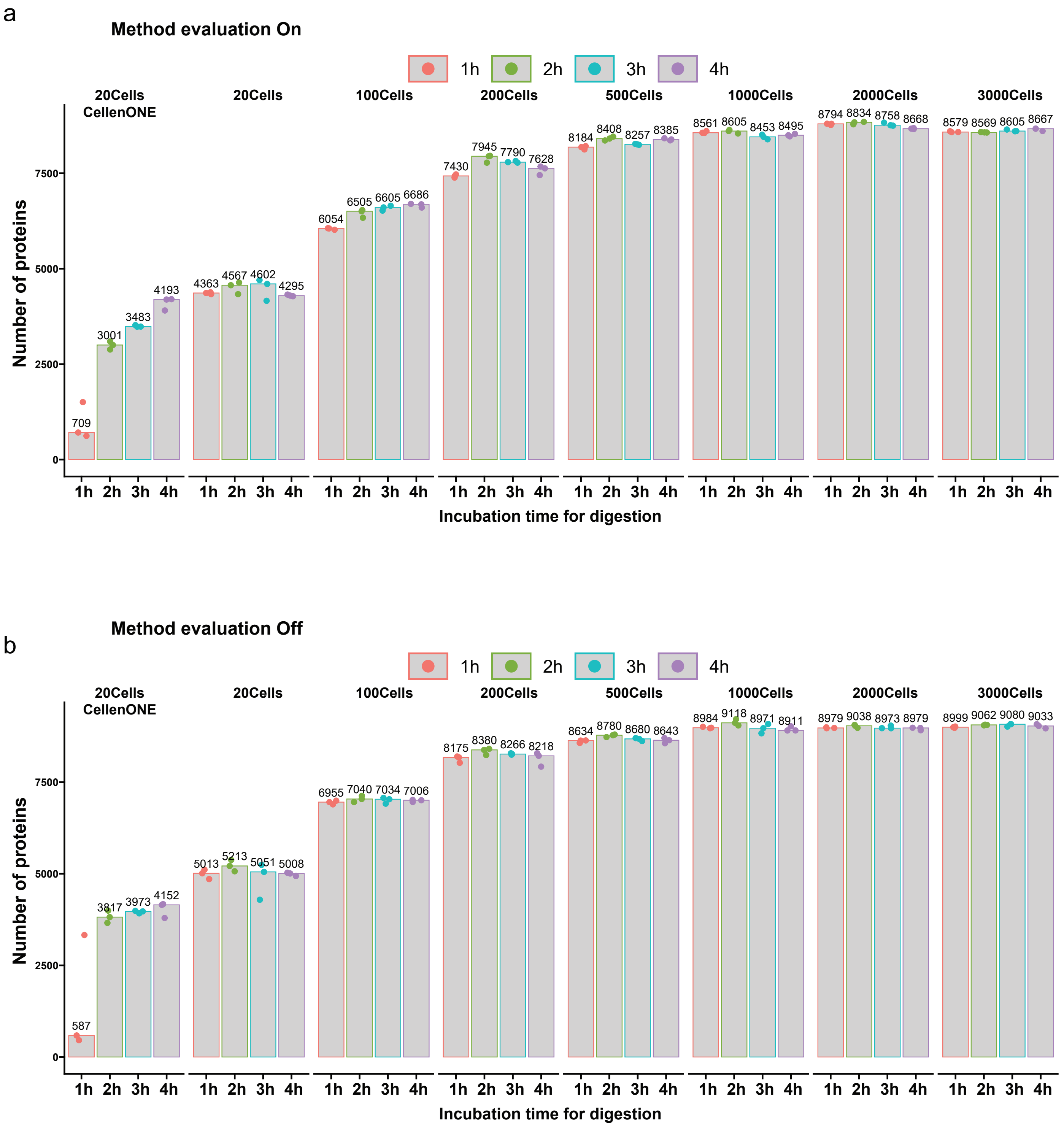

Supplementary Figure 1| Identification count of proteins across varying cell quantities using the One-Tip method. (**a**) With the 'Method Evaluation' option in Spectronaut enabled. (**b**) With the 'Method Evaluation' option in Spectronaut disabled. All samples underwent digestion times ranging from 1 to 4 hours. N=3 samples for each condition. Source data are provided as a Source Data file.

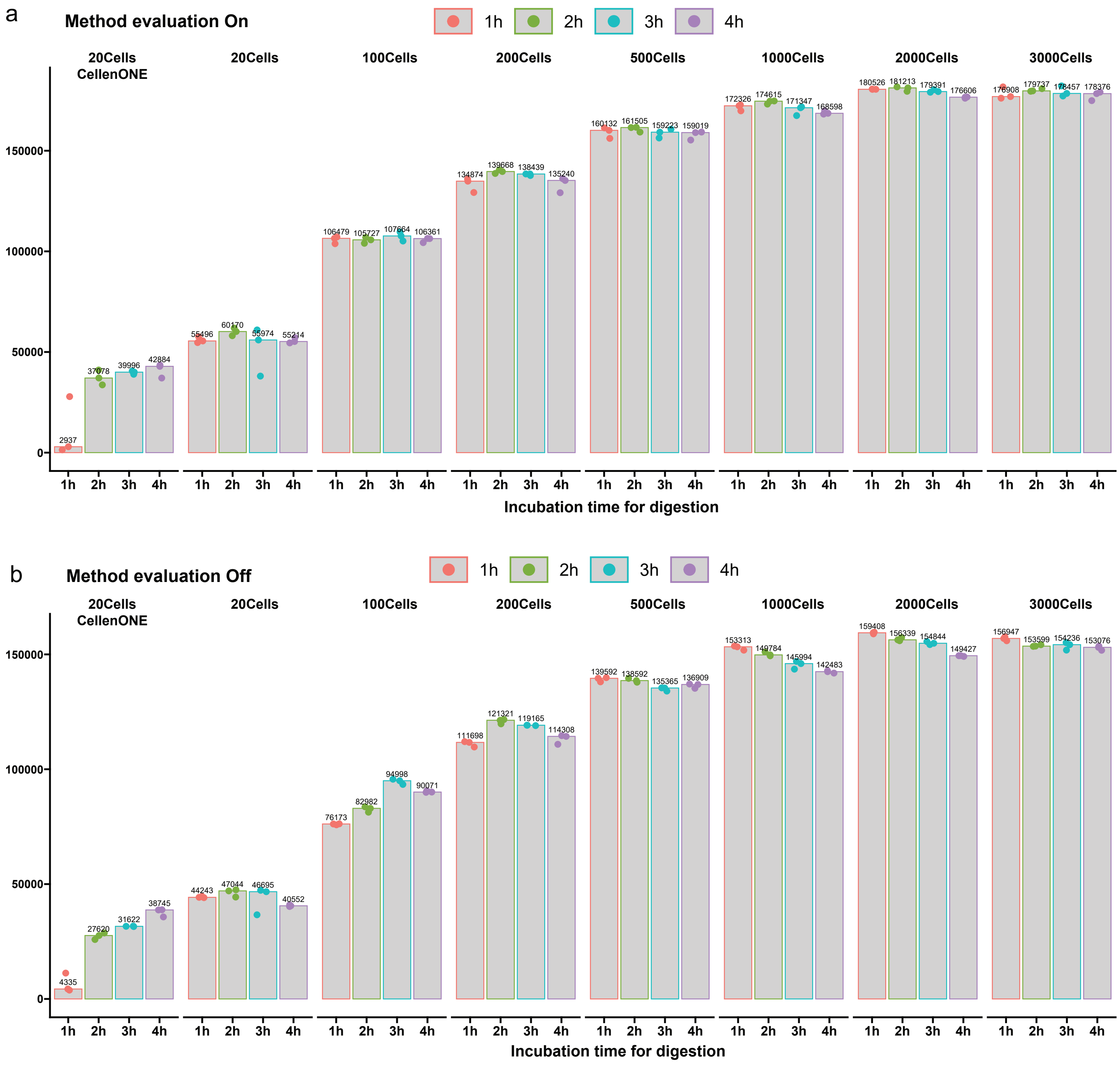

Supplementary Figure 2 | Identification count of peptides across varying cell quantities using the One-Tip method. (**a**) With the 'Method Evaluation' option in Spectronaut enabled. (**b**) With the 'Method Evaluation' option in Spectronaut disabled. All samples underwent digestion times ranging from 1 to 4 hours. N= 3 samples for each condition. Source data are provided as a Source Data file.

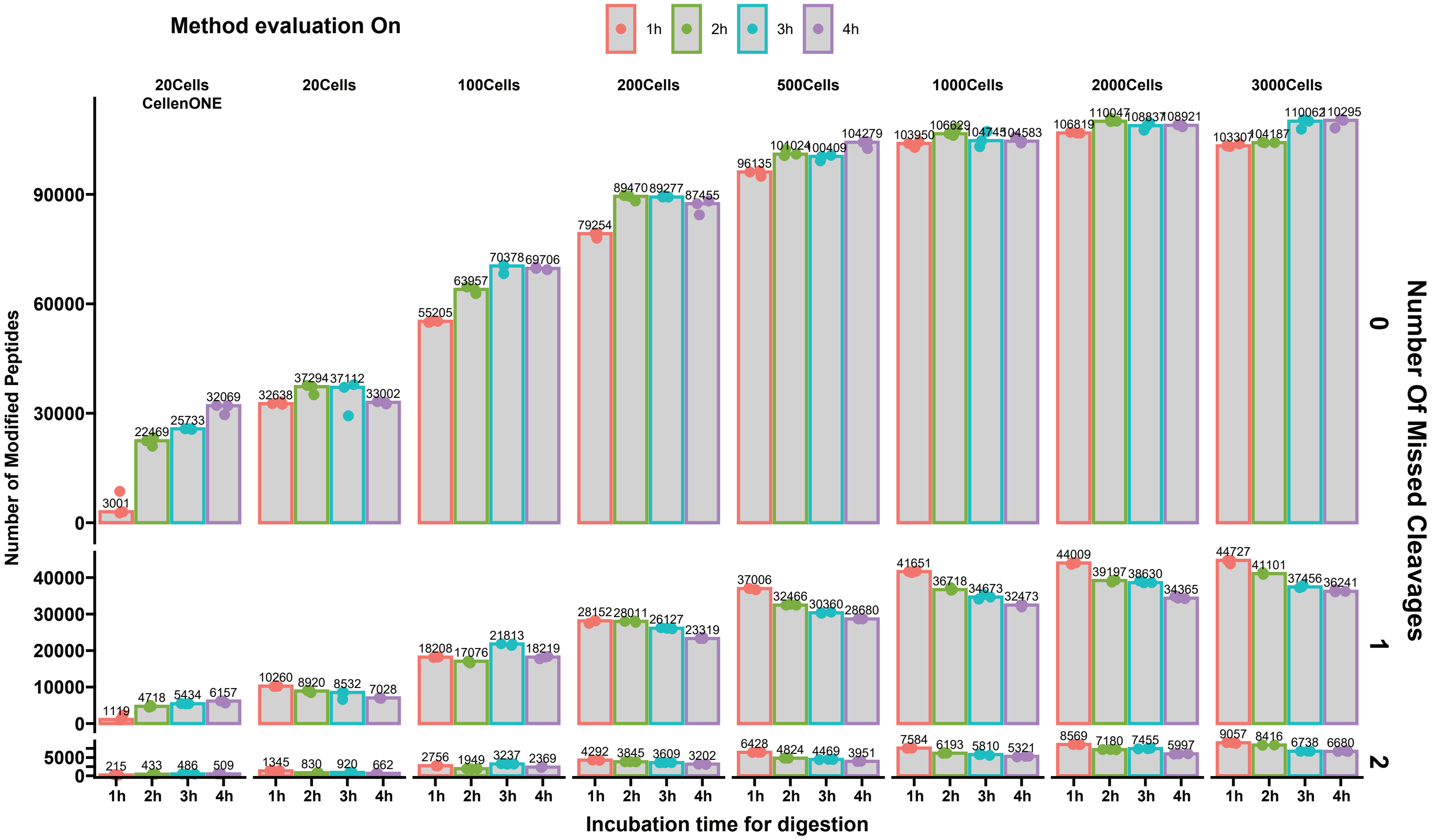

Supplementary Figure 3 | Count of peptides with differing numbers of missed cleavages, analyzed with the 'Method Evaluation' option in Spectronaut enabled. The digestion duration varied from 1 to 4 hours. N=3 samples for each condition. Source data are provided as a Source Data file.

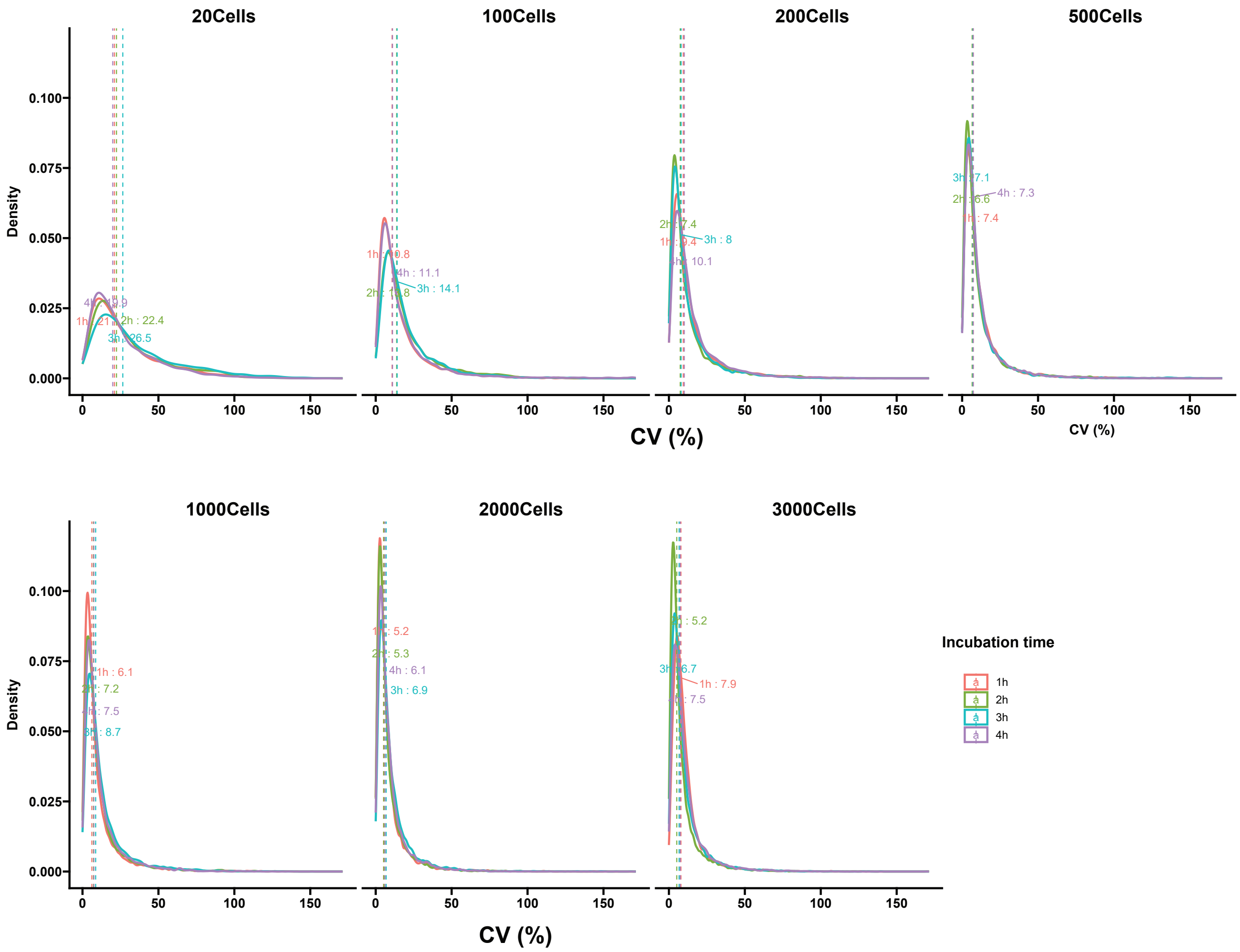

Supplementary Figure 4 | Distribution of coefficients of variation (CV) across different cell quantities processed via the One-Tip method. Digestion periods spanned 1 to 4 hours, and median CV values are annotated. Source data are provided as a Source Data file.

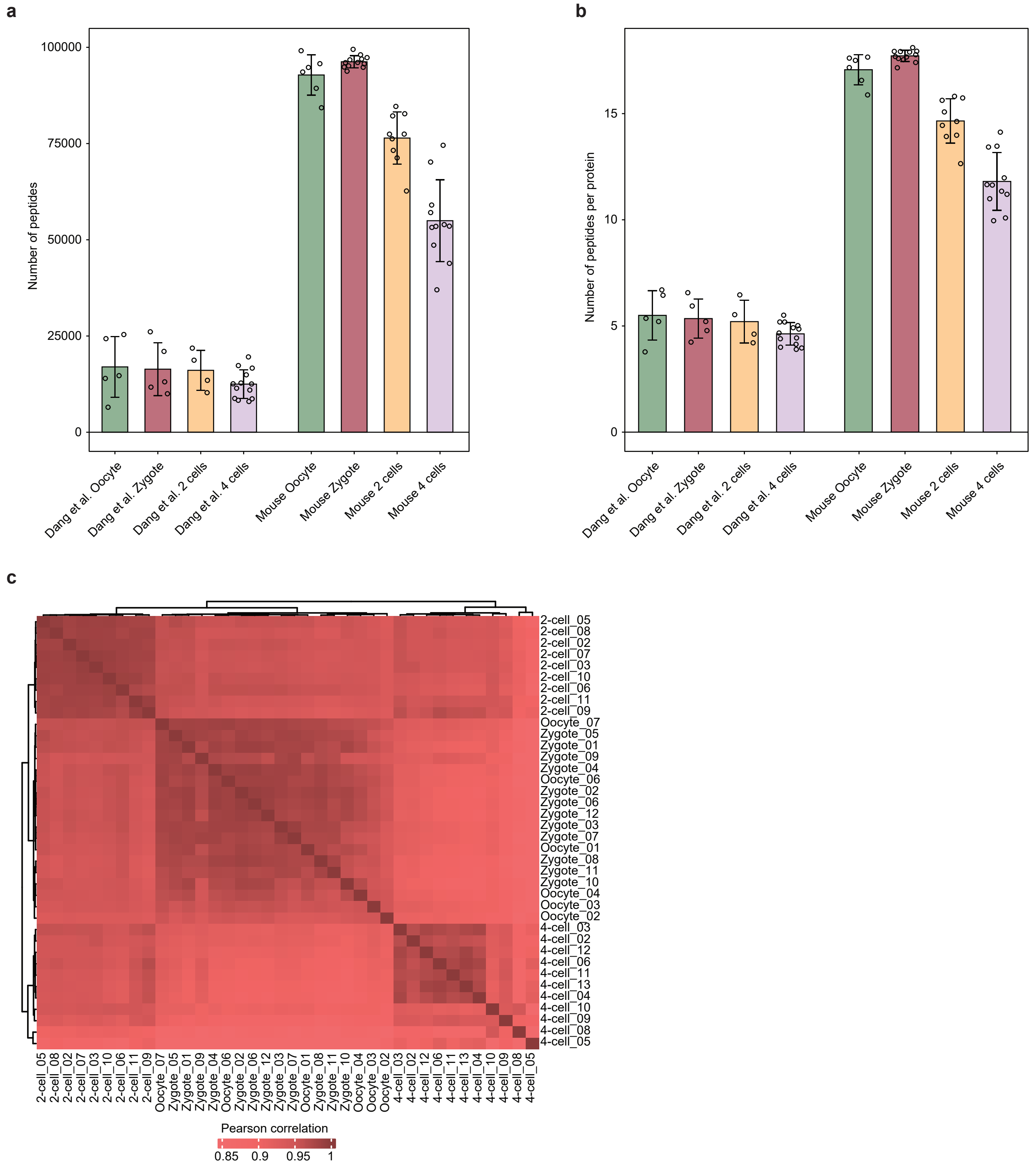

Supplementary Figure 5 | Number of peptides, correlation of protein intensities between samples and summed protein intensities. (**a**) Number of peptides per sample in Dang et al. study and our study. (**b**) Same with the number of peptides per protein. Error bars represent ± the standard deviation of the mean. (**c**) Unsupervised hierarchical clustering using canberra and ward.D2 methods of the Pearson correlation of normalized protein abundances between each sample. Number of biological replicates: n=6 for oocyte, n=12 for zygote, n=9 for 2-cell stage and n=11 for 4-cell stage. Source data are provided as a Source Data file.

.
