# Supplementary material for: One-Tip enables comprehensive proteome coverage in minimal cells and single zygotes
Source: Nat Commun. 2024 Mar 20;15:2474. doi: 10.1038/s41467-024-46777-9 (PMC10951212; doi:10.1038/s41467-024-46777-9)
Supplement: Supplementary file 2 — Reporting Summary [file 41467_2024_46777_MOESM2_ESM.pdf]

nature portfolio

Corresponding author(s): Jesper Velgaard Olsen

Last updated by author(s): Feb 28, 2024

Reporting Summary

Nature Portfolio wishes to improve the reproducibility of the work that we publish. This form provides structure for consistency and transparency in reporting. For further information on Nature Portfolio policies, see our Editorial Policies and the Editorial Policy Checklist.

Statistics

For all statistical analyses, confirm that the following items are present in the figure legend, table legend, main text, or Methods section.

n/a	Confirmed	
☐	☒	The exact sample size (*n*) for each experimental group/condition, given as a discrete number and unit of measurement
☐	☒	A statement on whether measurements were taken from distinct samples or whether the same sample was measured repeatedly
☐	☒	The statistical test(s) used AND whether they are one- or two-sided<br>*Only common tests should be described solely by name; describe more complex techniques in the Methods section.*
☐	☒	A description of all covariates tested
☐	☒	A description of any assumptions or corrections, such as tests of normality and adjustment for multiple comparisons
☐	☒	A full description of the statistical parameters including central tendency (e.g. means) or other basic estimates (e.g. regression coefficient) AND variation (e.g. standard deviation) or associated estimates of uncertainty (e.g. confidence intervals)
☐	☒	For null hypothesis testing, the test statistic (e.g. *F*, *t*, *r*) with confidence intervals, effect sizes, degrees of freedom and *P* value noted<br>*Give P values as exact values whenever suitable.*
☒	☐	For Bayesian analysis, information on the choice of priors and Markov chain Monte Carlo settings
☐	☒	For hierarchical and complex designs, identification of the appropriate level for tests and full reporting of outcomes
☒	☐	Estimates of effect sizes (e.g. Cohen's *d*, Pearson's *r*), indicating how they were calculated

Our web collection on statistics for biologists contains articles on many of the points above.

Software and code

Policy information about availability of computer code

Data collection	Mass spectrometry data was acquired using the Orbitrap Astral MS, using Thermo Tune software (version: 0.4 or higher).
Data analysis	Proteomics data was analyzed using Spectronaut (v18)

For manuscripts utilizing custom algorithms or software that are central to the research but not yet described in published literature, software must be made available to editors and reviewers. We strongly encourage code deposition in a community repository (e.g. GitHub). See the Nature Portfolio guidelines for submitting code & software for further information.

Data

Policy information about availability of data

All manuscripts must include a data availability statement. This statement should provide the following information, where applicable:

- Accession codes, unique identifiers, or web links for publicly available datasets
- A description of any restrictions on data availability
- For clinical datasets or third party data, please ensure that the statement adheres to our policy

The mass spectrometry proteomics data generated in this study have been deposited to ProteomeXchange Consortium (http://proteomecentral.proteomexchange.org) via the PRIDE partner repository with dataset identifier PXD044991 (https:/ https://www.ebi.ac.uk/pride/archive/projects/PXD044991).

Research involving human participants, their data, or biological material

Policy information about studies with human participants or human data. See also policy information about sex, gender (identity/presentation), and sexual orientation and race, ethnicity and racism.

Reporting on sex and gender	We only used blood samples from two anonymous male donors for technical tests. And thus, sex and gender were not considered in this study.
Reporting on race, ethnicity, or other socially relevant groupings	We only used blood samples from 2 anonymous male donors for technical tests. And thus, race, ethnicity, or other socially relevant groupings are irrelevant to this study.
Population characteristics	We only used blood samples from 2 anonymous male donors for technical tests. And thus population characteristic is irrelevant to this study.
Recruitment	Participants were randomly selected and the recruitment has no impact on the study since we only used the blood samples for technical purposes.
Ethics oversight	The study was performed in accordance with the guidelines of the Faculty of Health and medical Sciences of the University of Copenhagen.

Note that full information on the approval of the study protocol must also be provided in the manuscript.

Field-specific reporting

Please select the one below that is the best fit for your research. If you are not sure, read the appropriate sections before making your selection.

- [x] Life sciences
- [ ] Behavioural & social sciences
- [ ] Ecological, evolutionary & environmental sciences

For a reference copy of the document with all sections, see nature.com/documents/nr-reporting-summary-flat.pdf

Life sciences study design

All studies must disclose on these points even when the disclosure is negative.

Sample size	For proteomics experiments involving statistical analysis, we used at least biological triplicates to gain appropriate power for the discovery of true positive events. The sample sizes were chosen based on similar studies published previously (Kitata et al., 2021; Bekker-Jensen et al., 2020; Leutert et al., 2019).
Data exclusions	For mouse embryo analysis, 3 samples high number of missing values were excluded from the hierarchical clustering and PCA analysis since we excluded proteins that were not quantified in all samples and this would result in too few proteins present in the overall analysis and compromise its quality. This correspond to 1 oocyte, 1 cell of 2-cell stage and 1 cell of 4-cell stage.
Replication	All attempt at replicating the findings were successful.
Randomization	No randomization was performed for technical benchmark. Mouse samples were randomized before injection.
Blinding	Researchers were not blinded to performed the experiments reported in this work.<br>This was required as to keep a strict overview of mass spectrometry method selection.

Reporting for specific materials, systems and methods

We require information from authors about some types of materials, experimental systems and methods used in many studies. Here, indicate whether each material, system or method listed is relevant to your study. If you are not sure if a list item applies to your research, read the appropriate section before selecting a response.

Materials & experimental systems

n/a	Involved in the study	
x		Antibodies
	x	Eukaryotic cell lines
x		Palaeontology and archaeology
	x	Animals and other organisms
x		Clinical data
x		Dual use research of concern
x		Plants

Methods

n/a	Involved in the study	
x		ChIP-seq
x		Flow cytometry
x		MRI-based neuroimaging

Eukaryotic cell lines

Policy information about cell lines and Sex and Gender in Research

Cell line source(s)	HeLa cells (ATCC CCL-2).
Authentication	HeLa cells were authenticated by the supplier by STR profiling.
Mycoplasma contamination	All cell lines tested negative for mycoplasma contamination.
Commonly misidentified lines (See ICLAC register)	No commonly identified cell lines were employed in this study.

Animals and other research organisms

Policy information about studies involving animals; ARRIVE guidelines recommended for reporting animal research, and Sex and Gender in Research

Laboratory animals	Prepubescent (4-week old) C57BL/6NRj females (mus musculus) were used in this experiment.
Wild animals	No wild animals were used in this study.
Reporting on sex	Sex was not considered in the study design.
Field-collected samples	No field-collected samples were used in this study.
Ethics oversight	This study complies with all relevant ethical regulations. Animal work was conducted according to license no. 2021-15-0201-00851, approved by the Danish National Animal Experiments Inspectorate, and performed according to national and local guidelines.

Note that full information on the approval of the study protocol must also be provided in the manuscript.

Plants

Seed stocks	*Report on the source of all seed stocks or other plant material used. If applicable, state the seed stock centre and catalogue number. If plant specimens were collected from the field, describe the collection location, date and sampling procedures.*
Novel plant genotypes	*Describe the methods by which all novel plant genotypes were produced. This includes those generated by transgenic approaches, gene editing, chemical/radiation-based mutagenesis and hybridization. For transgenic lines, describe the transformation method, the number of independent lines analyzed and the generation upon which experiments were performed. For gene-edited lines, describe the editor used, the endogenous sequence targeted for editing, the targeting guide RNA sequence (if applicable) and how the editor was applied.*
Authentication	*Describe any authentication procedures for each seed stock used or novel genotype generated. Describe any experiments used to assess the effect of a mutation and, where applicable, how potential secondary effects (e.g. second site T-DNA insertions, mosiacism, off-target gene editing) were examined.*
